# Prediction of severe pancreatitis in a population with low atmospheric oxygen pressure

**DOI:** 10.1038/s41598-022-21789-x

**Published:** 2022-11-14

**Authors:** Germán Londoño-Ruiz, Camilo Ramírez-Giraldo, Andrés Vesga-Rosas, Felipe Vargas-Barato

**Affiliations:** 1 Hospital Universitario Mayor-Méderi, Calle 24 #29-45, Bogotá, Colombia; 2 Universidad del Rosario, Bogotá, Colombia

**Keywords:** Pancreatic disease, Biliary tract disease

## Abstract

To establish the severity of pancreatitis, there are many scoring systems, the most used are the Marshall and APACHE II systems, each one has advantages and disadvantages; but with good relation regarding mortality and prediction of complications. In populations with low barometric pressures produced by a decrease in atmospheric pressure, there is a decrease in partial pressure of oxygen, in these cases scores which take arterial oxygen partial pressure as one of their variables, may be overestimated. A diagnostic trial study was designed to evaluate the performance of APACHE II, Marshall and BISAP in a city 2640 m above sea level. A ROC analysis was performed to estimate the AUC of each of the scores, to evaluate the performance in predicting unfavorable outcomes (defined as the need for percutaneous drainage, surgery, or mortality) and a non-parametric comparison was made between the AUC of each of the scores with the DeLong test. From January 2018 to December 2019, data from 424 patients living in Bogota, with a diagnosis of gallstone pancreatitis was collected consecutively in a hospital in Bogota, Colombia. The ROC analysis showed AUC for predicting adverse outcomes for APACHE II in 0.738 (95% CI 0.647–0.829), Marshall in 0.650 (95% CI 0.554–0.746), and BISAP in 0.744 (95% CI 0.654–0.835). The non-parametric comparison to assess whether there were differences between the different AUC of the different scores showed that there is a statistically significant difference between Marshall and BISAP AUC to predict unfavorable outcomes (p=0.032). The mortality in the group of patients studied was 5.8%. We suggest the use of BISAP to predict clinical outcomes in patients with a diagnosis of biliary pancreatitis in populations with decreased atmospheric pressure because it is an easy-to-use tool and does not require arterial oxygen partial pressure for its calculation.

## Introduction

Acute pancreatitis is an inflammatory disorder of the pancreas^1^; it is a common pathology, the incidence varies between 4.9 and 73.4 cases per 100,000 worldwide^2^. This incidence is increasing, and it is associated to a high economic burden, it depends on different countries, their health care system and the degree of severity^3^.

In our institution more than 90% of acute pancreatitis are of biliary origin. Although most cases of pancreatitis are self-limited in terms of the patient's systemic involvement and a non-complicated development, up to 20% can present local complications and up to 5% mortality depending on the degree of severity^4^. For this reason, the Atlanta consensus recommends the classification of this pathology as mild, moderately severe or severe according to the appearance and persistence of organic failure, and this in turn is related to its mortality^5^. To establish this severity, there are many scoring systems, the most used are the Marshall and APACHE II (Acute Physiology and Chronic Health Evaluation II) systems, each one has advantages and disadvantages; but with good relation regarding mortality and prediction of complications^6,7^.

Many of these classification systems are complex, use multiple values that are not readily available, and may delay an appropriate approach in patients with acute pancreatitis. The BISAP (Bedside Index for Severity in Acute Pancreatitis) system was proposed as a system that uses five variables to define the severity of acute pancreatitis; it has demonstrated a good predictive ability for unfavorable outcomes associated with easy calculation and as gained ample acceptance among surgical groups around the world^8,9^.

**Figure 1:**
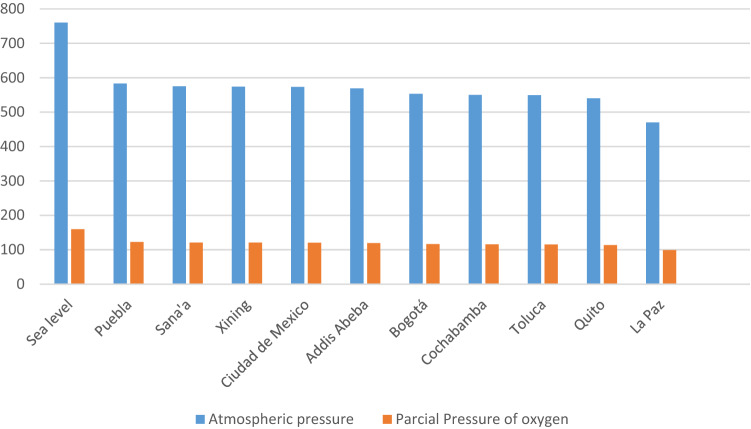
Atmospheric pressure and partial pressure of oxygen according to the altitude where the population is located.

However, in populations with low barometric pressures produced by a decrease in atmospheric pressure, there is a decrease in partial pressure of oxygen, this happens in populations like La Paz, Quito, Toluca, Cochabamba, Bogota, Addis Ababa, Mexico City, Xining, Sana'a, Puebla, among others (Fig. 1); in these cases, scores such as Marshall and APACHE, which take arterial oxygen partial pressure as one of their variables, may be overestimated, as defined normal values for the relationship between arterial oxygen partial pressure and inspired oxygen fraction (PaFi) may not be equivalent. This could be a potential advantage of the BISAP system, as it does not consider arterial oxygen partial pressure, therefore, it is independent of atmospheric pressure and could be better correlated with disease severity by making a risk assessment of unfavorable clinical outcomes less excessive than the other scores^10,11^.

The objective of this study is to evaluate the performance of APACHE II, Marshall and BISAP to predict unfavorable outcomes in a city 2640 m above sea level.

## Methods

From January 2018 to December 2019, data from 424 patients living in Bogota, with a diagnosis of gallstone pancreatitis, per the Atlanta Consensus criteria^5^, was collected consecutively in a hospital in Bogota, Colombia. The variables were collected in an anonymous database. The study was performed according to the list of essential items reporting diagnostic accuracy studies^12^.

Patients under 18 years of age, with non-biliary pancreatitis, cases of chronic or recurrent pancreatitis, from another city and patients with absence of all variables to calculate severity scores were excluded.

All patients were managed according to the institutional protocol based on international guidelines, with a step-up approach^9,13,14^, and patients with pancreatitis classified as severe according to APACHE II or Marshall were admitted to the Intensive Care Unit^5^.

The predictive ability of the APACHE II, Marshall, and BISAP scores for unfavorable outcomes defined as the need for percutaneous drainage, surgery, or mortality was evaluated. The cut-off points with which we consider the severity of pancreatitis are shown in Table 1 for the different scores.

**Table 1:** Cut-off points to define the severity of pancreatitis.

Score	Points	Severity
Marshall	0–1	Mild
	≥ 2	Severe
APACHE II	0–7	Mild
	≥ 8	Severe
BISAP	0–2	Mild
	≥ 3	Severe

This study did not represent any intervention on the patients and all the information was collected retrospectively from their medical records. For this reason, it is considered at risk-free study according to Colombian law. The confidentiality of individual data was preserved. Upon admission to the institution, patients gave a written informed consent to use their clinical information for research purposes. The study protocol and statistical analysis was approved by the research committee of the Hospital Universitario Mayor-Méderi and by the ethics committee of Universidad del Rosario (number DVO005 1120-CV1218).

### Statistical analysis

A description of the demographic variables collected the clinical results, and the predictive scores was made. Categorical variables were described in rates, and continuous variables were described in means. A univariate analysis (chi square test and Mann–Whitney test) was then performed to evaluate differences between demographic and clinical variables according to the severity of pancreatitis considering a statistically significant difference (p < 0.05).

A ROC analysis was performed to estimate the area under the curve (AUC) of each of the scores, to evaluate the performance in predicting unfavorable outcomes. A non-parametric comparison was made between the AUC of each of the scores with the DeLong test^15^.

The entire analysis was performed in SPSS^®^26, considering a statistically significant p < 0.05.

### Ethical standards

Ethical compliance with the Helsinki Declaration, current legislation on research Res. 008430-1993 and Res. 2378-2008 (Colombia) and the International Committee of Medical Journal Editors (ICMJE) were ensured under our Ethics and Research Institutional Committee (IRB) approval. Informed consent was filled out as required for the execution of this study.

## Results

**Figure 2:**
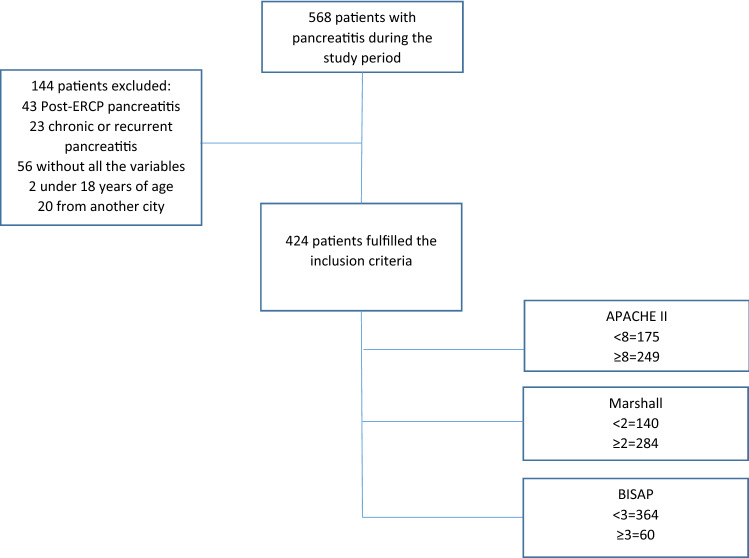
Flowchart of the study selection process.

A total of 424 patients were included in the study, the flow chart shows the selection process (Fig. 2). 43 patients with post-endoscopic retrograde cholangiopancreatography (ERCP) pancreatitis were excluded. There were no alcohol-induced acute pancreatitis and no lipidemia-induced acute pancreatitis because in our population they are infrequent etiologies and without surgical follow-up. All patients were calculated the different scores at the time of admission.

The patients evaluated had a mean age of 60.69 ± 19.94 and there was a female predominance (62.74%), the other characteristics can be observed in Table 2.

**Table 2:** Demographic and clinical characteristics.

	N (%)
Age (mean ± SD) (years)	60.69 ± 19.94
**Gender**	
Female	266 (62.74)
Male	158 (37.26)
**Co-morbidity**	
Hypertension	83 (19.58)
Diabetes mellitus	37 (8.73)
COPD	33 (7.78)
History of malignant disease	32 (7.55)
Others	127 (29.95)
PaFi	275.75 ± 64.75
APACHE II (mean ± SD) (points)	8.44 ± 4.23
Marshall (mean ± SD) (points)	1.92 ± 1.09
BISAP (mean ± SD) (points)	1.35 ± 1,11
Local complications	60 (14.15)
Days in hospital (mean ± SD) (days)	10.38 ± 8.95
Days in ICU (mean ± SD) (days)	2.82 ± 5.27
Percutaneous drainage	6 (1.42)
Surgery	18 (4.25)
Mortality	25 (5.9)

In the univariate analysis according to the severity of the different scores, we found that there were differences in mortality if the APACHE II or BISAP score were severe, while Marshall showed no differences. On the other hand, we found differences in the surgical requirement for the three scores and for the percutaneous drainage requirement there were no statistically significant differences (Table 3).

**Table 3:** Characteristics according to severity scores.

	APACHE II		Marshall		BISAP	
	< 8 (N = 175)	≥ 8 (N = 249)	< 2 (N = 140)	≥ 2 (N = 284)	< 3 (N = 364)	≥ 3 (N = 60)
Age (mean ± SD) (years)	46.00 ± 17.62*	71.01 ± 14.19*	48.79 ± 19.66*	66.56 ± 17.32*	58.01 ± 19.87*	76.95 ± 10.43*
**Gender (%)**						
Female	124 (70.86)*	142 (57.03)*	102 (72.86) *	164 (57.75) *	234 (64.29)	32 (53.33)
Male	51 (29.14)*	107 (42.97)*	38 (27.14)*	120 (42.25)*	130 (35.71	28 (46.67)
**Co-morbidity (%)**						
Hypertension	24 (13.71)*	59 (23.69)*	19 (13.57)*	64 (22.54)*	66 (18.13)	17 (28.33)
Diabetes mellitus	7 (4.00)*	30 (12.05)*	7 (5.00)	30 (10.56)	32 (8.79)	5 (88.33)
COPD	6 (3.43)*	27 (10.84)*	6 (4.29)	27 (9.51)	24 (6.59)*	9 (15.00)*
History of malignant disease	8 (4.57)	24 (9.64)	11 (7.86)	21 (7.39)	22 (6.04)*	10 (16.67)*
Others	26 (14.86)*	101 (40.56)*	26 (18.57)*	101 (35.56)*	99 (27.2)*	28 (46.67)*
PaFi	299.81 ± 51.78*	258.84 ± 67.63*	327.45 ± 40.02*	250.26 ± 59.21*	279.90 ± 53.56*	250.60 ± 107.98*
**Local complications (%)**						
Yes	16 (9.14)*	44 (17.67)*	8 (5.71)*	52 (18.31)*	47 (12.91)	13 (21.67)
No	159 (90.86)	205 (82,33)	132 (94.29)*	232 (81.69)*	317 (87.09)	47 (78.33)
Days in hospital (mean ± SD) (days)	7.21 ± 4.70*	12.62 ± 10.45*	8.02 ± 6.74*	11.55 ± 9.67*	9.54 ± 7.49*	15.53 ± 14.08*
Days in ICU (mean ± SD) (days)	0.64 ± 2.09*	4.36 ± 6.21*	0.9 ± 2.1*	3.77 ± 6.05*	2.46 ± 5.23*	5.00 ± 5.01*
**Percutaneous drainage (%)**						
Yes	1 (0.57)	5 (2.01)	0 (0.00)	6 (2.11)	5 (1.37)	1 (1.67)
No	174 (99.43)	244 (97.99)	140 (100)	278 (97.89)	359 (98.63)	59 (98.33)
**Surgery (%)**						
Yes	3 (1.71)*	15 (6.02)*	1 (0.71)*	17 (5.99)*	11 (3.02)*	7 (11.67)*
No	172 (98.29)*	234 (93.98)*	139 (99.29)*	267 (94.01)*	353 (96.98)*	53 (88.33)*
**Mortality (%)**						
Alive	171 (97.71)*	228 (91.57)*	135 (96.43)	264 (92.96)	348 (95.6)*	51 (85.00)*
Died	4 (2.29)*	21 (8.43)*	5 (3.57)	20 (7.04)	16 (4,40)*	9 (15.00)*

*p-values < 0.05 were statistically significant.

**Figure 3:**
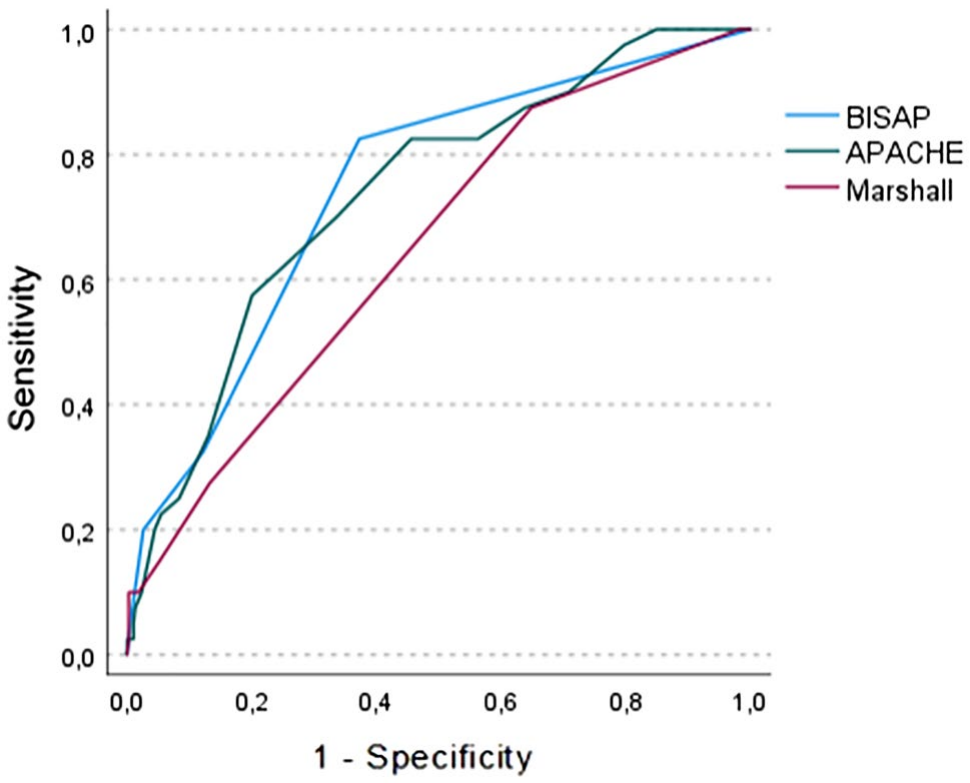
ROC curves for predicting surgical outcomes for different scores.

The ROC analysis showed AUC for predicting unfavorable outcomes for APACHE II in 0.738 (95% CI 0.647–0.829), Marshall in 0.650 (95% CI 0.554–0.746), and BISAP in 0.744 (95% CI 0.654–0.835) (Fig. 3).

The non-parametric comparison to assess whether there were differences between the different AUC of the different scores showed that there is a statistically significant difference between Marshall and BISAP AUC to predict unfavorable outcomes (p=0.032).

## Discussion

We included 424 patients, with biliary pancreatitis seen during 2018 and 2019; as reported in the literature, with biliary etiology as the main cause of acute pancreatitis. The mean age of the patients was 60.69 years, in relation to previous studies that have shown that pancreatitis affects people of productive age^16^. As in other studies, a tendency was found to affect females more (62.74%), possibly because women have a higher incidence of benign biliary disease^17^.

The mortality reported in the literature for pancreatitis may be 1% in general^6^, although directly related to severity, in its severity forms it may reach up to 30%^18^; in our case the mortality in the group of patients studied was 5.8%; this is in relation to the fact that cases of severe pancreatitis were higher in this population and that our center is a regional reference center.

We found that 284 (66.98%) of the pancreatitis cases were severe according to the Marshall, 249 (58.72%) were severe according to the APACHE II, and 60 (14.15%) were severe according to the BISAP. This result is notorious, as the data found in our population show a much higher proportion of pancreatitis classified as severe with APACHE II and Marshall scores, when compared to other studies^2,19^; the reason for these findings is related to lower arterial oxygen partial pressure when compared other populations due to the altitude above sea level, so there is an overestimation of severity. In contrast, with BISAP, the proportion of pancreatitis classified as severe is more similar to that reported^19^.

When we assess the predictability of the three scores to predict adverse outcomes, we find that Marshall performance is below expectations and below what has been observed in other studies^20^. The same may be related to decreased PaFi because when the PaFi is below 300 it should be assigned 2 points in the Marshall score, this implies that this patient is already classified as severe. Morevoer, APACHE II has a better performance in the prediction of unfavorable outcomes, even though the PaFi relationship is also within its variables, this is, because it is not the only variable that must be altered so that the score is greater than or equal to 8 which is the score that defines pancreatitis as severe, but other parameters must be altered. In the case of BISAP we observed that it is the best performing score to predict unfavorable outcomes in our population with an AUC 0.744, probably because it does not take PaFi into account within its variables. So, in our midst it is an alternative to consider.

When assessing whether there were differences between AUC the three scores to predict unfavorable outcomes, we can show that there is a statistically significant higher performance of BISAP over Marshall, and there are no statistically significant differences between BISAP and APACHE II.

Overestimation of the severity of acute pancreatitis leads to higher hospital costs and leads these patients to occupy beds in the intensive care unit unnecessarily, thus increasing the days of hospital stays, exposing patients to complications associated with hospitalization such as infections and thrombotic diseases.

The limitations of our study lie in its retrospective nature and that we only included patients with biliary pancreatitis, which are predominant in our context, pancreatitis with other kinds of etiology should be evaluated in another study.

These findings, allow us to suggest that the BISAP score be used for stratification of severity in all patients with acute biliary pancreatitis in populations where PaFi may be altered due to decreased partial pressure of oxygen while other studies are being conducted evaluating others scores with the PaFi adjusted to the partial pressure of oxygen according to atmospheric pressure. In addition, BISAP is easy to establish and has been evaluated for the prediction of unfavorable outcomes being an alternative comparable to APACHE II and superior to other scores^19,21,22^.

## Conclusions

We suggest the use of BISAP to predict clinical outcomes in patients with a diagnosis of biliary pancreatitis in populations with decreased atmospheric pressure because it is an easy-to-use tool and does not require arterial oxygen partial pressure for its calculation.

## Supplementary Information

Supplementary Information.

## Data Availability

All data generated or analysed during this study are included in this published article [and its supplementary information files].

## Abbreviations

APACHE II: Acute Physiology and Chronic Health Evaluation II
AUC: Area under the curve
BISAP: Bedside Index for Severity in Acute Pancreatitis
ERCP: Post-endoscopic retrograde cholangiopancreatography
PaFi: Relationship between arterial oxygen partial pressure and inspired oxygen fraction
